# Prophylactic and therapeutic effect of AZT/3TC in RT-SHIV infected Chinese-origin rhesus macaques

**DOI:** 10.1186/1742-6405-11-12

**Published:** 2014-03-04

**Authors:** Wei Wang, Nan Yao, Zhe Cong, Hong Jiang, Chuan Qin, Qiang Wei

**Affiliations:** 1 Comparative Medicine Center, Peking Union Medical College (PUMC), Beijing 100021, China; 2 Institute of Laboratory Animal Science, Chinese Academy of Medical Sciences(CAMS), Beijing 100021, China; 3 Key Laboratory of Human Diseases Comparative Medicine, Ministry of Health, Beijing 100021, China; 4 Key Laboratory of Human Diseases Animal Models, State Administration of Traditional Chinese Medicine, Beijing 100021, China

**Keywords:** RT-SHIV, Animal model, Chinese-origin rhesus macaque, Post-exposure prophylaxis, Therapy

## Abstract

**Background:**

The precise efficacy of nucleoside analogue reverse-transcriptase inhibitors (NRTIs) in preventing and inhibiting virus replication remains unknown in RT-SHIV infected Chinese-origin rhesus macaques (Ch RM).

**Findings:**

Ch RM were inoculated intravenously with 200 TCID_50_ RT-SHIV and treated by gavage with NRTIs (20 mg AZT and 10 mg 3TC twice per day) for four consecutive weeks beginning at one hour, on day 217 or 297 post inoculation, respectively. Treatment with AZT/3TC inhibited transiently RT-SHIV replication during chronic infection, but did not significantly affect peripheral blood CD4^+^ T cells in macaques. Treatment with AZT/3TC at 1 hour post infection prevented RT-SHIV infection in two out of four animals during the 120-day observation period.

**Conclusions:**

Therefore, the Ch RM model with RT-SHIV infection can be used to evaluate the efficacy of new NRTIs.

## Findings

Animal models, such as non-human primate (NHP) models, are very useful for the preclinical evaluation of anti-viral drugs and microbicides [1-3]. Due to a low sensitivity of simian immunodeficiency virus (SIV) to non-nucleoside reverse transcriptase inhibitors (NNRTIs) and some nucleoside reverse transcriptase inhibitors (NRTIs), especially for post-exposure prophylaxis (PEP), a number of RT-SHIV strains have been constructed to evaluate the activity of HIV-specific drugs and microbicides both *in vitro* and in macaques [4-8]. RT-SHIV carry HIV-1 reverse transcriptase (RT) gene, which is the target of NNRTIs and NRTIs, suitable for evaluating the efficacy of HIV RT inhibitors in macaques [1,9]. Up to now, RT-SHIV is commonly used to study the effect of highly active antiretroviral therapy (HAART) and antiviral resistance in India origin rhesus macaques (In RM) [1], pigtailed monkey [9,10], and Chinese-origin rhesus macaques (Ch RM) [11]. An increasing body of evidence demonstrates that Ch RM are useful in evaluating the pathogenesis, vaccine, and therapeutic strategies for HIV/AIDS infection [11]; however, Ch RM are different from In RM in viral infection, immunological response, and host genetic background [12,13]. It is important to evaluate the virus and infectious character in Ch RM. For example, Pal et al. characterized vaginal transmission of RT-SHIV in Ch RM and showed that RT-SHIV isolates were sensitive to RT inhibitors *in vitro*[11]. However, the precise efficacy of RT inhibitors in preventing and inhibiting virus replication remains unknown in RT-SHIV/TC infected Ch RM model. In this study, we choose two representative NRTIs, zidovudine (AZT) and lamivudine (3TC), to study their effect on preventing and inhibiting virus replication in Ch RM.

Twelve healthy Ch RMs at 3 years of age (weighing at 4 kg, equal number of males and females) were born and housed in a specific pathogen free (SPF) facility at Institute of Laboratory Animal Science (ILAS), Chinese Academy of Medical Sciences. All naïve animals were negative for tests of simian type D retroviruses (SRV), simian T cell leukemia virus-1 (STLV), simian immunodeficiency virus (SIV), monkey B virus (BV), and tubercle bacillus (TB). Animal care was compliant with the Institute of Laboratory Animal Science Guidelines for the Care and Use of Laboratory Animals (est. 2006), and the experimental protocol was approved by the Animal Research and Care Committee of ILAS (Additional file 1: Supplementary Materials and Methods). Ch RM were inoculated intravenously with 200 TCID_50_ (3.35 × 10^5^ copies/mL) cell-free RT-SHIV, in which the RT gene of SIVmac239 was replaced with that of HIV-1 clone HXBc2 [5,14,15] (Additional file 1: Supplementary Materials and Methods). This dose was similar to the amount of virus in a contaminated needle (estimated 25 μl of blood from a patient with high viral load) through which individuals obtained a primary infection, and was lower than other studies in In RM [1]. Those macaques were randomized and provided with sterile water during the observation period as the controls [16], or treated by oral gavage twice per day with both 20 mg AZT (Sigma, St. Louis, USA) and 10 mg 3TC (Sigma, calculated, according to body surface area, similar to that of human use) [17] beginning at one hour after virus inoculation for four consecutive weeks as the PEP group. These drug powders were weighed and mixed prior to delivery. Four additional macaques were treated by oral gavage with the same dose of AZT and 3TC twice per day beginning on day 217 or 297 post virus inoculation, when the viremia had reached a plateau, as the therapeutic group. Following inoculation and treatment, the contents of plasma virus and the numbers of peripheral CD4^+^ T cells were determined longitudinally by quantitative RT-PCR (Additional file 1: Supplementary Materials and Methods) and flow cytometry analysis [18] (Additional file 1: Supplementary Materials and Methods), respectively.

**Figure 1:**
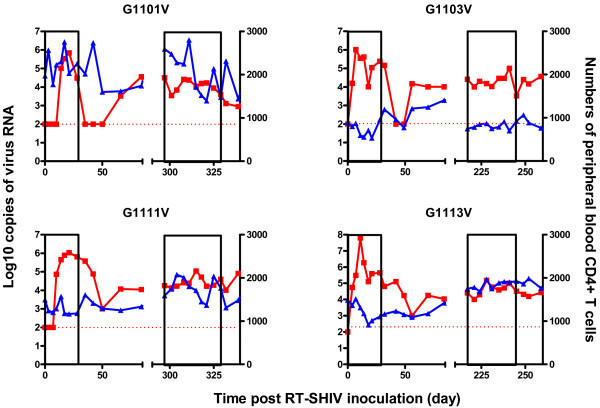
**The levels of plasma viral loads and peripheral blood CD4**^**+ **^**T cells in individual control macaques.** Data are expressed as the mean number of copies of viral RNA (red line) and peripheral blood CD4^+^ T cells (blue line) of individual macaques through the observation period. The areas of pockmarks show the time periods with AZT/3TC or water gavage.

**Figure 2:**
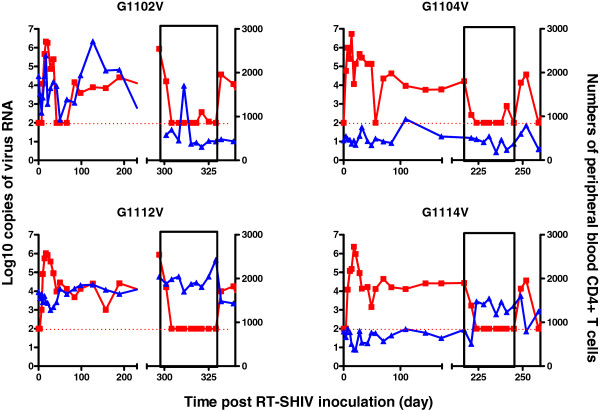
**The levels of plasma viral loads and peripheral blood CD4**^**+ **^**T cells in individual macaques treated with AZT/3TC on day 297 or 217 post inoculation.** Data are expressed as the mean number of copies of viral RNA (red line) and peripheral blood CD4^+^ T cells (blue line) of individual macaques through the observation period. The areas of pockmarks show the time periods with AZT/3TC or water gavage.

**Figure 3:**
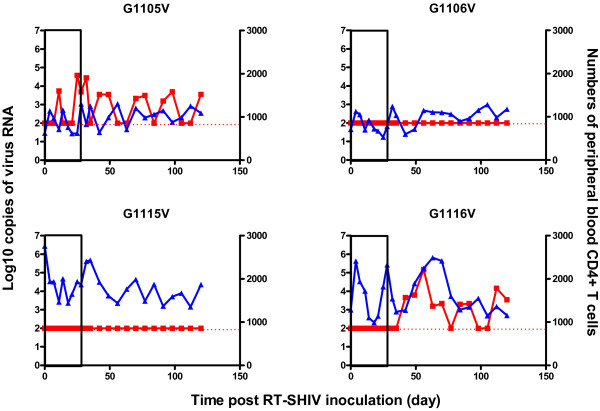
**The levels of plasma viral loads and peripheral blood CD4**^**+ **^**T cells in individual macaques treated with AZT/3TC one hour post inoculation.** Data are expressed as the mean number of copies of viral RNA (red line) and peripheral blood CD4^+^ T cells (blue line) of individual macaques through the observation period. The areas of pockmarks show the time periods with AZT/3TC or water gavage.

As expected, plasma RT-SHIV-specific RNA was detected in the control group of macaques and reached a peak of 0.69 ~ 6.30 × 10^6^ copies per ml at 1 or 3 weeks post inoculation (Figure 1), consistent with a previous report that RT-SHIV effectively infects Ch RM [11]. Eight to 12 weeks after infection, RT-SHIV viremia reached a plateau and maintained a high level of 1.0 to 3.5 × 10^4^ copies per ml in the control group of macaques throughout the observation period. Furthermore, treatment with a combination of AZT with 3TC starting on day 297 or 217 post-infection significantly reduced the levels of plasma viral loads in these macaques (G1102V, G1104V, G1112V and G1114V), and the levels of viremia in those experimental monkeys were lower than that of the detection threshold (virus RNA load <100 copies/mL) at most time points post drug treatment (Figure 2). These data indicated that treatment with both AZT and 3TC inhibited RT-SHIV replication in macaques. More importantly, treatment with both AZT and 3TC one hour after inoculation dramatically prevented and inhibited RT-SHIV replication in macaques. Evidentially, there was no detectable viremia and viral RNA in three macaques throughout the drug treatment period, and only moderate levels of viremia were detected in another macaque (G1105V) at the phase of drug treatment. One (G1116V) out of three treated monkeys had viral rebound after the end of drug treatment. Similar patterns of provirus DNA were detected in the PBMC of the different groups of monkeys (data not shown). Interestingly, the numbers of peripheral blood CD4^+^ T cells at the end of the observation period were similar to that at the baseline, except for one macaque (G1102V) that received AZT/3TC on day 297 post inoculation with nearly 65% reduced numbers of peripheral blood CD4^+^ T cells (Figures 2 and 3). Furthermore, there were no obvious clinical symptoms in the infected animals, including G1102V, and these animals remained healthy during the experimental period. Therefore, treatment with a combination of AZT and 3TC prevented and inhibited RT-SHIV replication, but had little effect on the numbers of peripheral blood CD4^+^ T cells in macaques.

This study provided evidence to demonstrate that treatment with AZT/3TC prevented and inhibited RT-SHIV replication in a Ch RM model of AIDS. A number of RT-SHIV strains, in which the RT was replaced with the RT from an HIV-1 clone, have been constructed to evaluate the efficacy and safety of HIV-specific NNRTIs and NRTIs both *in vitro* and in macaques [1,5,6,19]. These stains of viruses are not only highly sensitive to HIV-1 RT-specific NNRTIs, but also to a variety of NRTIs and protease inhibitors, which inhibit virus replication [1,15]. Hence, RT-SHIV is an appropriate virus for challenge to evaluate the efficacy of anti-HIV NNRTIs and NRTIs. Although In RM macaque is commonly used in biological studies as non-human primate, the limited numbers of In RM macaques available for research have hampered our studies to understand the AIDS pandemic. In addition, studies of non-human primate in a single model of animals may lead to biased results and misleading findings [20]. The Ch RM have a big population available for research and represent a potential resource of animals for expanding the current research efforts. In this study, we infected Ch RM with 200 TCID_50_ RT-SHIV and observed that treatment with both AZT and 3TC one hour post inoculation prevented RT-SHIV replication in two out of four macaques and treatment with the same drugs at peak infection inhibited virus replication in four macaques. These data indicated that RT-SHIV was sensitive to NRTIs in Ch RM. Conceivably, treatment with these medicines may effectively prevent HIV replication and AIDS development if a natural infection occurs with a dose of HIV. Our data are consistent with a previous report that prophylactic treatment with a single substance post exposure reduces the probability of an infection [21]. Our results suggest that the animal model infected with RT-SHIV can be used to evaluate new NRTIs for the treatment and prevention of AIDS.

In summary, our data indicate that AZT and 3TC treatment post inoculation of RT-SHIV can prevent and inhibit RT-SHIV replication in Ch RM. Therefore, the RT-SHIV/Ch RM model may be valuable to evaluate NRTIs.

## Abbreviations

NRTIs: Nucleoside analogue reverse-transcriptase inhibitors; Ch RM: Chinese-origin rhesus macaque; NHP: Non-human primate; SIV: Simian immunodeficiency virus; NNRTIs: Non-nucleoside reverse transcriptase inhibitors; PEP: Post-exposure prophylaxis; RT: Reverse transcriptase; HIV: Human immunodeficiency virus; HAART: Highly active antiretroviral therapy; In RM: India origin rhesus macaque; AZT: Zidovudine; 3TC: Lamivudine; SPF: Specific pathogen free; ILAS: Institute of Laboratory Animal Science; SRV: Simian type D retroviruses; STLV: Simian T cell leukemia virus-1; BV: Monkey B virus; TB: Tubercle bacillus.

## Competing interests

The author declares that they have no competing interests.

## Authors’ contributions

WW wrote the manuscript, designed the study and analyzed the data. NY participated in the collection of data of CD4^+^ T cell count. ZC participated in the collection of data of plasma viral load and analyzed the data. HJ participated in the manipulation of animal. CQ and QW participated in the design of the study. All authors have read and approved the final manuscript.

## Supplementary Material

Additional file 1 Supplementary Materials and Methods. Click here for file
