# Supplementary material for: Prophylactic and therapeutic effect of AZT/3TC in RT-SHIV infected Chinese-origin rhesus macaques
Source: AIDS Res Ther. 2014 Mar 4;11:12. doi: 10.1186/1742-6405-11-12 (PMC4016570; doi:10.1186/1742-6405-11-12)
Supplement: Additional file 1 — Supplementary Materials and Methods. [file 1742-6405-11-12-S1.docx]

Additional file 1

**Materials and Methods**

**Animals and animal care**

Twelve healthy, 3 years old Ch RMs were used in this study, equal numbers of male and female. The monkeys were born and housed in a specific pathogen free (SPF) facility at Institute of Laboratory Animal Science (ILAS), Chinese Academy of Medical Sciences. All animals tested negative for simian type D retroviruses, simian T cell leukemia virus-1, and SIV prior to study participation. The Institutional Animal Care and Use Committee (IACUC) of Institute of Laboratory Animal Science, Chinese Academy of Medical Sciences (ILAS, CAMS) approved all macaque procedures described (protocol permit number ILAS-VL-2010-006). ILAS facilities used in this study are fully accredited by the Association for Assessment and Accreditation of Laboratory Animal Care International (AAALAS). This study was carried out in strict accordance with the recommendations in the Guide for the Care and Use of Laboratory Animals of the Institute of Laboratory Animal Science (est. 2006) and with the recommendations of the Weatherall report; “The use of non-human primates in research”. All procedures were performed under anesthesia using ketamine hydrochloride. Because the experiments described here involved a virus that may cause an incurable disease, such as AIDS, discomfort, stress and pain may occur, so animals were closely monitored and observed for development of disease at least twice daily. If the animals are determined to be under stress or in discomfort, appropriate anesthetics and/or analgesics are administered as directed by the clinical veterinary staff.

**Virus, infections and dosing**

RT-SHIV was constructed with a SIVmac239 with the Reverse Transcriptase replaced with that of HIV-1 clone HXBc2, and Contains a T to C substitution at position 8 of the SIV tRNA primer binding site that improves replication [[1-3](#_ENREF_1)]. The virus strain (11342) was obtained through the AIDS Research and Reference Reagent Program, Division of AIDS, NIAID, NIH.

All of Ch RMs were inoculated intravenously with 200 TCID_50_ (3.35×10^5^ copies/mL) cell-free RT-SHIV. Among them, eight macaques were randomized and provided with sterile water during the corresponding drug period as the controls (G1101V, G1103V, G1111V and G1113V) [[4](#_ENREF_4)], or treated by oral gavage with combination of 20mg AZT and 10 mg 3TC thrice daily beginning at one hour after virus inoculation for four consecutive weeks as the PEP group (G1105V, G1106V, G1115V and G1116V). Four additional macaques were treated by oral gavage with the same dose of AZT and 3TC daily beginning on day 217 (G1104V and G1114V) or 297 (G1102V and G1112V) post virus inoculation. All animals were observed until four weeks after stop of treatment.

**Plasma viral RNA analysis**

The plasma virus load was quantified by quantitative real-time reverse transcription polymerase chain reaction (qRT-PCR) using the LightCycler 2.0 instrument (Roche, Indianapolis, IN, USA). The 20 μL reactions contained LightCycler FastStart DNA Master SYBR Green 4, 2-f and 2-r primers (5’-GTA ACT ATG TCC ACC TGC CAT TA-3’ and 5’-CAG CCT CCT CGT TTA TGA TGT-3’), MgCl_2_, and plasma RNA sample. The RT reaction was carried out by serial incubation at 37°C for 15 min and 50°C for 30 min. The amplification reaction was carried out by 95°C for 10 min (initial activation) followed by 45 cycles (amplification) of 94°C for 15 sec, 56°C for 20 sec, and 72°C for 30 sec. For each run, standard curves were generated by amplifying serial dilutions of a reference SIV gag (GI: 530752666). Copy numbers were determined by interpolation onto the standard curve with the LightCycler software, version 3.5.

**Flow cytometric assay**

Peripheral blood samples from individual monkeys were evaluated for the percentages of CD4^+^ and CD8^+^ T cells by flow cytometry (all reagents and instrumentation from BD Biosciences). Briefly, blood samples (100 μl) were incubated with fluorochrome-conjugated antibodies (PerCP-CD3 (clone SP34-2), FITC-CD4 (clone L200), and PE-CD8 (clone RPA-T8)) for 30 min in the dark, and the red blood cells were lysed by incubating with the FACS Lysing Buffer. After being washed, the cells were analyzed by flow cytometry on a BD FACSCanto flow cytometer, and at least 20,000 events were analyzed using the FACSDiva 6.1 software.

Reference

1. Uberla K, Stahl-Hennig C, Bottiger D, Matz-Rensing K, Kaup FJ, Li J, Haseltine WA, Fleckenstein B, Hunsmann G, Oberg B, et al.: **Animal model for the therapy of acquired immunodeficiency syndrome with reverse transcriptase inhibitors.** *Proceedings of the National Academy of Sciences of the United States of America* 1995, **92:**8210-8214.

2. North TW, Van Rompay KK, Higgins J, Matthews TB, Wadford DA, Pedersen NC, Schinazi RF: **Suppression of virus load by highly active antiretroviral therapy in rhesus macaques infected with a recombinant simian immunodeficiency virus containing reverse transcriptase from human immunodeficiency virus type 1.** *Journal of virology* 2005, **79:**7349-7354.

3. Soderberg K, Denekamp L, Nikiforow S, Sautter K, Desrosiers RC, Alexander L: **A nucleotide substitution in the tRNA(Lys) primer binding site dramatically increases replication of recombinant simian immunodeficiency virus containing a human immunodeficiency virus type 1 reverse transcriptase.** *Journal of virology* 2002, **76:**5803-5806.

4. Le Grand R, Vaslin B, Larghero J, Neidez O, Thiebot H, Sellier P, Clayette P, Dereuddre-Bosquet N, Dormont D: **Post-exposure prophylaxis with highly active antiretroviral therapy could not protect macaques from infection with SIV/HIV chimera.** *AIDS* 2000, **14:**1864-1866.
